# Taxonomy of myid bivalves from fragmented brackish-water habitats in India, with a description of a new genus *Indosphenia* (Myidae, Myoidea, Myidae)

**DOI:** 10.3897/zookeys.799.25843

**Published:** 2018-11-28

**Authors:** P. Graham Oliver, Anders Hallan, P.R. Jayachandran, Philomina Joseph, V.F. Sanu, S. Bijoy Nandan

**Affiliations:** 1 National Museum of Wales, Cathays Park, Cardiff CF10 3NP, UK National Museum of Wales Cardiff United Kingdom; 2 Malacology Division, Australian Museum Research Institute, Sydney, Australia Australian Museum Research Institute Sydney Australia; 3 Department of Marine Biology, Microbiology and Biochemistry, School of Marine Sciences, Cochin University of Science &Technology, Lakeside campus, Cochin 682016, India Cochin University of Science &Technology Cochin India

**Keywords:** Anatomy, Bivalvia, brackish waters, COI, India, morphology, taxonomy

## Abstract

A group of small bivalves inhabiting Indian brackish-water estuaries and lagoons (known locally as backwaters), variously assigned to *Corbula*, *Cuspidaria*, and *Sphenia*, are reviewed and, based on shell characters, shown to be congeneric. Molecular (COI) and morphological data indicate that this group belongs to the family Myidae. Furthermore, the combined data suggest that these Indian myids are a sister taxon of the genus *Sphenia*. The Indian material studied herein exhibits a functional morphology typical of infaunal bivalves, whereas typical *Sphenia* are nestling and epibyssate. A new genus, *Indosphenia*, is thus erected for the Indian group and includes five species, one of which is named in this study.

*Indospheniakayalum* Oliver, Hallan & Jayachandran, **gen. et sp. n.** is described from the Cochin Backwater on the western coast of India. *Cuneocorbulacochinensis* (Preston, 1916) is transferred to *Indosphenia*. Additionally, the west coast taxa *I.abbreviata* (Preston, 1907), *I.abbreviatachilkaensis* (Preston, 1911) and *I.sowerbyi* (EA Smith, 1893) are recognised herein. *Corbulaalcocki* Preston, 1907, *Corbulagracilis* Preston, 1907, *Corbulacalcaria* Preston, 1907 and *Corbulapfefferi* Preston, 1907 are placed in synonymy with *I.abbreviata*, and *Cuspidariaannandalei* Preston, 1915 is synonymised with *I.abbreviatachilkaensis*.

## Introduction

**Figure 1.**
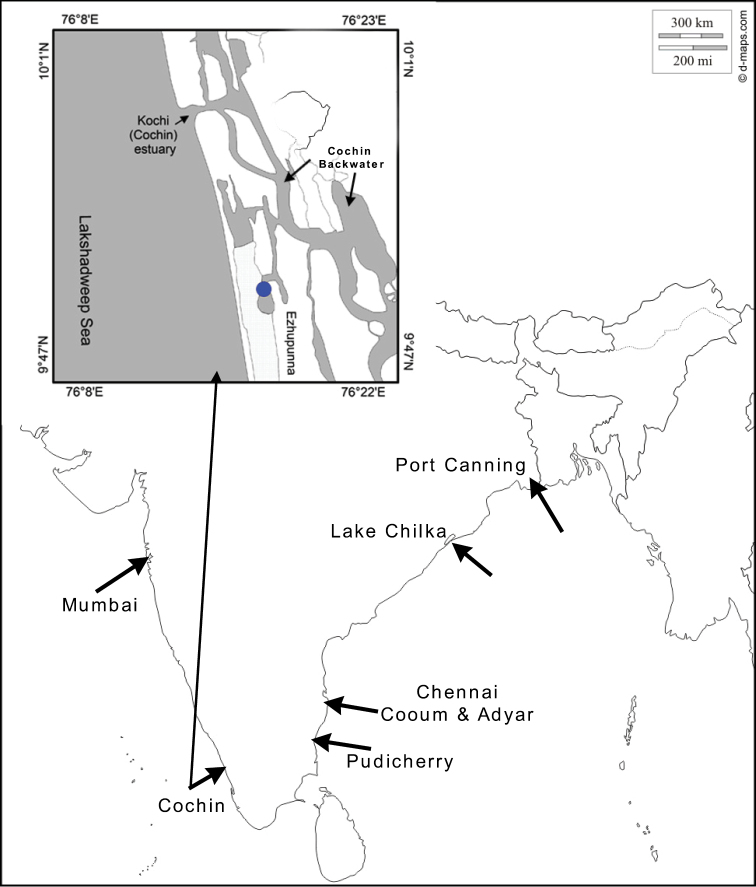
Map of India showing locations of sample sites. Insert showing detailed location of type locality of *Indospheniakayalum* within the Cochin Backwater.

Extensive estuarine and lagoon systems are found around both the eastern and western coasts of India (Fig. 1). The malacofauna was extensively investigated in the early twentieth century and many new taxa were published by HB Preston (1907, 1911, 1915, 1916), see Table 1.Preston described rather similar morphotypes as separate species from the isolated and geographically separated brackish water environments such as the Gangetic Delta, Lake Chilka and Cochin Backwater. The taxonomy of many bivalve species described by Preston has been confusing, at least in part because Preston did not describe details of the hinge. Consequently, some species were described under *Cuspidaria* while others under *Corbula*, groups that are not closely related and only superficially similar with respect to their shell morphology. In some instances, the original generic placements remained unchallenged (Ramakrishna and Dey 2010) while others were changed (Oliver et al. 2016). Preston (1907) described five new species of *Corbula* from one single locality, but there was no indication of any habitat segregation or the degree of morphological variation. This led to an inconsistent situation in the subsequent literature as summarised below.

**Table 1.** List of species considered in this paper recorded from the Indian subcontinent.

Original combination	Name currently used in MolluscaBase (2018)	Type locality
*Spheniaperversa* (Blanford, 1867)	*Spheniaperversa* (Blanford, 1867)	Pegu, Irawady Delta, Myanmar
*Spheniasowerbyi* EA Smith, 1893	*Spheniasowerbyi* EA Smith, 1893	Pondicherry
*Corbulaabbreviata* Preston, 1907	*Potamocorbulaabbreviata* (Preston, 1907)	Port Canning
*Corbulaalcocki* Preston, 1907	*Potamocorbulaalcocki* (Preston, 1907)	Port Canning
*Corbulacalcaria* Preston, 1907	*Potamocorbulaabbreviata* (Preston, 1907)	Port Canning
*Corbulagracilis* Preston, 1907	*Potamocorbulaabbreviata* (Preston, 1907)	Port Canning
*Corbulapfefferi* Preston, 1907	*Potamocorbulaabbreviata* (Preston, 1907)	Port Canning
*Corbulachilkaensis* Preston, 1911	*Potamocorbulachilkaensis* (Preston, 1911)	Lake Chilka
*Cuspidariaannandalei* Preston, 1915	*Cuspidariaannandalei* Preston, 1915	Lake Chilka
*Cuspidariacochinensis* Preston, 1916	*Cuneocorbulacochinensis* (Preston, 1916)	Cochin Backwater

Ramakrishna and Dey (2010) synonymised *Corbulaalcocki* and *C.pfefferi* under *C.abbreviata* while retaining *C.calcarea* and *C.gracilis* as valid species; additionally, they transferred *C.chilkaensis* to *Cuspidaria*. Huber (2010) argued that all of the above species described by Preston in 1907 should be considered a single species, proposing the name *Potamocorbulaabbreviata* (Preston, 1907) be used. Subba Rao (2017) followed Huber (2010) and also placed *Cuspidariaannandalei* into synonymy with *Corbulachilkaensis* under *Potamocorbulachilkaensis*.

Bearing this taxonomic background in mind, in 2016, one of the present authors (Philomina Joseph) collected a sample of fragile, thin-shelled bivalves living among filamentous green algae, on a muddy substrate, in the upper brackish regions of the Cochin Backwater. This bivalve could not be identified from the available literature including the latest book on Indian bivalves by Subba Rao (2017). The aim of this research was primarily to identify this enigmatic bivalve, but as the authors undertook this work they realised that all the Indian brackish water taxa assigned to *Potamocorbula* are in fact members of the Myidae, and not the Corbulidae.

Two other species of Myidae have been recorded from Indian estuaries; *Spheniaperversa* was recorded from various localities on the coast of the Bay of Bengal by Subba Rao et al. (1992; 1995) and by Dey (2008). *Spheniasowerbyi*, which was described from the Chennai (Madras) region, has not been recorded in Indian taxonomic literature cited above, but was cited in an ecological paper on tidal pools near Mumbai (Satam and Deshmukh 2013). The myid fauna of the Indian lagoons and estuaries is summarised in Table 1 and it is this set of taxa that are reviewed in this paper.

## Materials and methods

Very little original material exists for those taxa described by Preston (1907, 1911, 1916), except for syntypes now held in the ZSI (Kolkata), NHMUK (London), and NMW (Cardiff). Access to these type specimens has been limited, and the co-joined valves of the few specimens made available have been deemed too fragile to separate and adequately examine. Fortunately, a relatively large number of shells from Port Canning (the type locality for Preston’s (1907) species) are present in the NHMUK, as is an extensive series of *Spheniasowerbyi* from Chennai. The only material available for molecular work was that recently collected from Cochin by P Joseph. Given that only one Indian species was available for DNA extraction the only aim of the molecular study was to support the family placement. The use of COI was adopted for this purpose and other genes were not analysed because this paper does not attempt a molecular phylogeny of the Myoida.

### Molecular analysis

Specimens for molecular examination were preserved in 100% ethanol. The ethanol- preserved samples were re-hydrated in sterile distilled water for 10–12 hours at ambient room temperature prior to DNA extraction. Genomic DNA was extracted from macerated muscle tissue using the DNeasy Blood & Tissue Kit (Qiagen) following the spin column protocol. The polymerase chain reaction (PCR) mixture consisted of 25 μL Master Mix (Takara Clontech EmeraldAmp® GT PCR Master Mix), 1 μL forward primer, 1 μL reverse primer, 8 μL template DNA, and 15 μL distilled deionised water. The amplification primers were LCO-1490 F (5'- GGTCAACAAATCATAAAGATATTGG-3') and HCO-2198 R (5'-TAAACTTCAGGGTGACCAAAAAATCA-3'), used for amplifying mitochondrial cytochrome c oxidase subunit I (mtCOI) gene sequences (Folmer et al. 1994).

Amplification was carried out in an Agilent thermal cycler (Sure cycler 8800). The amplification protocol followed a sequence of denaturation at 94 °C for 1 min., annealing at 37 °C for 2 mins and extension at 72 °C for 3 mins; 40 cycles were performed. Amplified products exhibiting distinct bands after agarose gel (1.2%) electrophoresis were purified and sent to SciGenom Labs (SciGenom Labs Pvt, Ltd. Ernakulam, India) for sequencing.

For the analysis, only the forward primer sequences were used. Sequences with a product length of 614–625 base pairs were obtained without any gaps or stop codons. The sequences thus obtained were assembled using BioEdit 7.0.9 (Hall 1999) and the alignment was performed using ClustalX (Thompson et al. 1997).

BioEdit v7.0.9 (Hall 1999) was used to compile sequences, which were subsequently aligned with MEGA7 (Kumar et al. 2016). Missing nucleotides in the alignment were substituted by N’s. A hierarchical likelihood test was performed in MEGA7 to identify the most suitable model for phylogenetic analysis.

**Figure 2.**
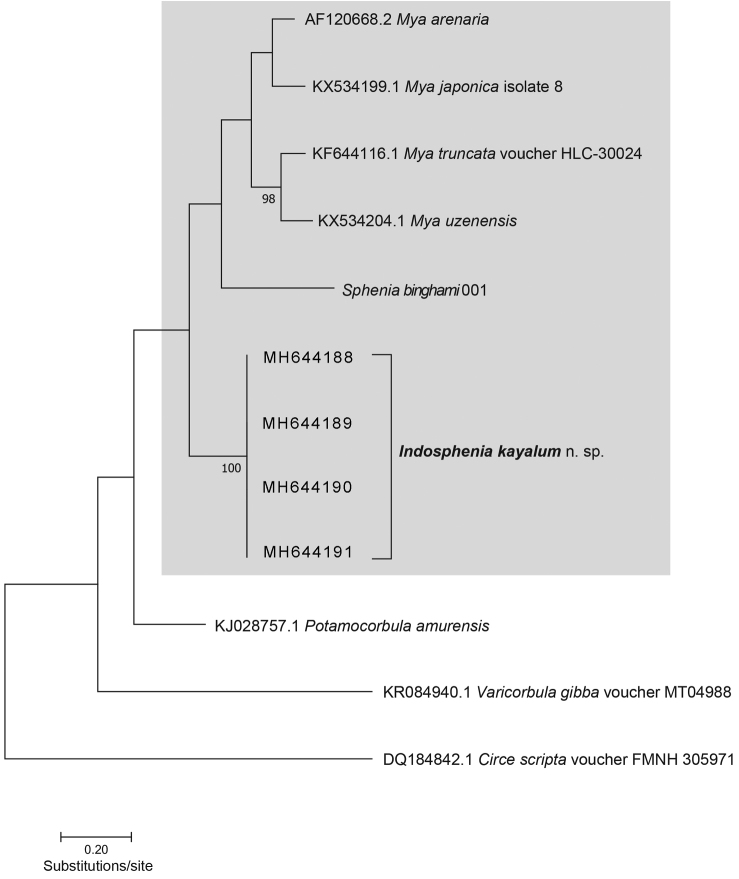
Phylogram showing Maximum Likelihood method based on the Hasegawa-Kishino-Yano model. The tree with the highest log likelihood (- 2576.44) is shown. The percentage of trees in which the associated taxa clustered together is shown next to the branches where such values exceed 95%. Initial tree(s) for the heuristic search were obtained automatically by applying Neighbor-Join and BioNJ algorithms to a matrix of pairwise distances estimated using the Maximum Composite Likelihood (MCL) approach, and then selecting the topology with superior log likelihood value. Grey area indicates Myidae.

MEGA7 was used also to conduct a Maximum Likelihood analysis with 1000 bootstrap repetitions. In all, six myid species plus three outgroup taxa (two corbulids plus the venerid *Circescripta* [Linnaeus, 1758]) were chosen for the molecular analysis. GenBank accession vouchers for these outgroup taxa are shown in the phylogram. The phylogram was generated using MEGA7. Sequences have been submitted to GenBank and their identification codes are attached to the phylogram in Fig. 2.

### Microscopy

Specimens were examined under a Leica MZ12 stereomicroscope and photographed with a Leica Z6 macroscope with Helicon Focus stacking software. Anatomical observations were made on formaldehyde-fixed specimens using the above microscopes; some specimens were stained in methylene blue to enhance contrasts, whereas some were fixed in Bouin’s solution. For scanning electron microscopy, specimens were dried and gold-coated prior to observation with a Jeol Neoscope. The shells were measured with an eyepiece graticule. The statistical analyses were applied from JMP™ statistical software.

### Specimens examined

Details of the specimens described in this study are given below for each taxon. Type material in the Zoological Survey of India was not studied directly because this institute will not lend material, and funds were not available to visit Kolkata. Syntypes of some species were available in British museums and original and subsequent illustrations were used. Attempts to borrow the material described as *Spheniasowerbyi* by Satam and Deshmukh (2013) were unsuccessful.

### Comparative material examined

*Spheniaperversa* 6 specimens, Kungkraben Bay, Thailand, Coll PG Oliver. 3 valves, Bombay, ex ME Deakin Coll., NHMUK1909.9.23.306-8.

*Spheniabinghami* 4 specimens, Pwlldu Bay, South Wales, coll. PG Oliver; many shells from Tenby and Weymouth.

*Spheniarueppelli* 1 shell, Yemen, Red Sea, NMW.Z.1995.008.11.

Spheniacf.rueppelli 12 shells, Karachi, NHMUK 20090386. *Myaarenaria* juvenile specimens from the British Isles, NMW.

*Myatruncata* juvenile specimens from the British Isles, NMW.

Institutional abbreviations:

**NMW/NMWZ** National Museum of Wales, Cardiff

**NHMUK** Natural History Museum, London

**ZSI** Zoological Survey of India, Kolkata

## Results

### Molecular Analysis

The evolutionary history was inferred by using the Maximum Likelihood method based on the Hasegawa-Kishino-Yano model (Hasegawa et al. 1985). The tree with the highest log likelihood (-2576.44) is shown in this study (Fig. 2). A discrete Gamma distribution was used to model evolutionary rate differences among sites (5 categories (+*G*, parameter = 0.4468)). The analysis involved 12 nucleotide sequences. Codon positions included were 1^st^+2^nd^+3^rd^+Noncoding. All positions containing gaps and missing data were eliminated. There were 401 positions in the final dataset.

Although based solely on a single gene and with a limited dataset, our phylogenetic analysis strongly suggests that the new species belongs in the family Myidae, thus corroborating the morphological evidence. We note that the bootstrap support throughout the phylogram is not strong overall (only two branches are statistically significant, see Fig. 2); however, this may be resolved in future analyses where additional genes more suited for elucidating deeper branches are used. *Mya* is recovered as monophyletic in this analysis and is sister to *Spheniabinghami*. *Mya* and *Sphenia* are joined as sister to the brackish-water taxon described in this study, in which four identical haplotypes are recovered in the phylogram.

### Morphology

**Figure 3.**
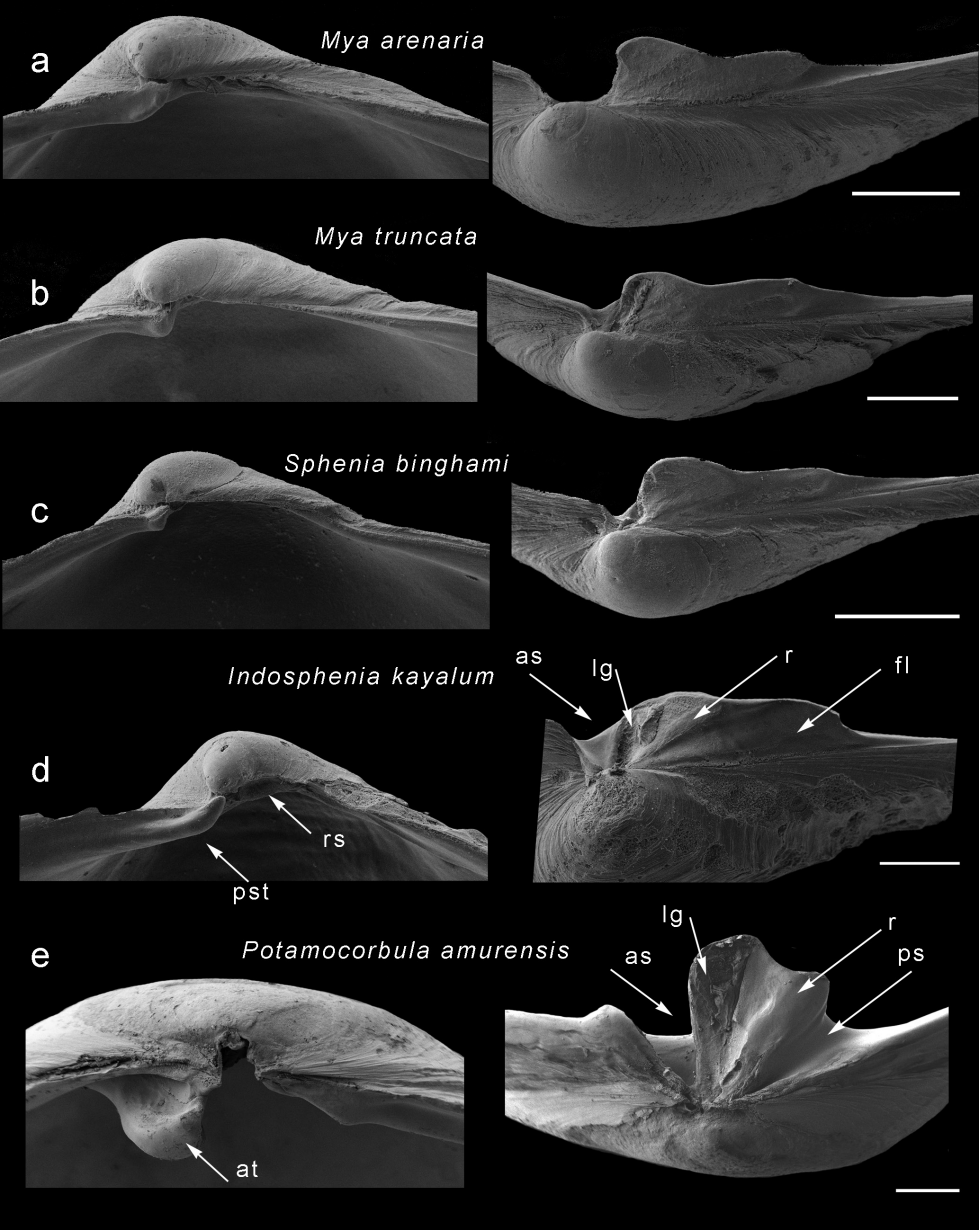
Scanning electron micrographs of hinges of myids and *Potamocorbula*. Left hand series are right valves, left hand series are left valves with the chondrophore viewed from above. **a***Myaarenaria***b***Myatruncata***c***Spheniabinghami***d***Indospheniakayalum***e***Potamocorbulaamurensis*. Scale bar: 500 µm. Abbreviations: as, anterior socket. at, anterior tooth. fl, posterior flange. lg, ligament plate. ps, posterior socket. pst, pseudotooth. r, ridge. rs, resilifer.

The hinge morphology of the five species examined in this study (*Spheniasowerbyi*, *Corbulaalcocki*, *Corbulagracilis*, *Cuspidariaannandalei* and the Cochin Backwater sample) is consistent with that of the Myidae, notably with *Myaarenaria*, *M.truncata*, and *Spheniabinghami*. Huber’s (2010) placement of these in *Potamocorbula* (family Corbulidae) can be dismissed through comparison of the hinge morphology (Fig. 3a–e). In *Potamocorbula* (Fig. 3e) and the similar *Lentidium*, there is a well-defined projecting cardinal tooth in the right valve. This tooth arises from below the hinge margin and fits into a deep socket in the right valve anterior to the chondrophore. In both corbulid genera, the chondrophore is heavy and relatively narrow. In the myids (Fig. 3a–d) there is a pseudo-tooth in the right valve, but this develops as a thickening of the hinge margin and is never as well-developed as the corresponding tooth in corbulids. The myid chondrophore, as seen in *Sphenia* (Fig. 3c, d) and juvenile *Mya*(Fig. 3a, b), has a posterior flange extending behind the ligament area. This suggests that most, if not all, of the Indian brackish water taxa assigned to *Corbula*, *Cuspidaria*, and *Sphenia* share more affinities with the Myidae than the Corbulidae.

**Figure 4.**
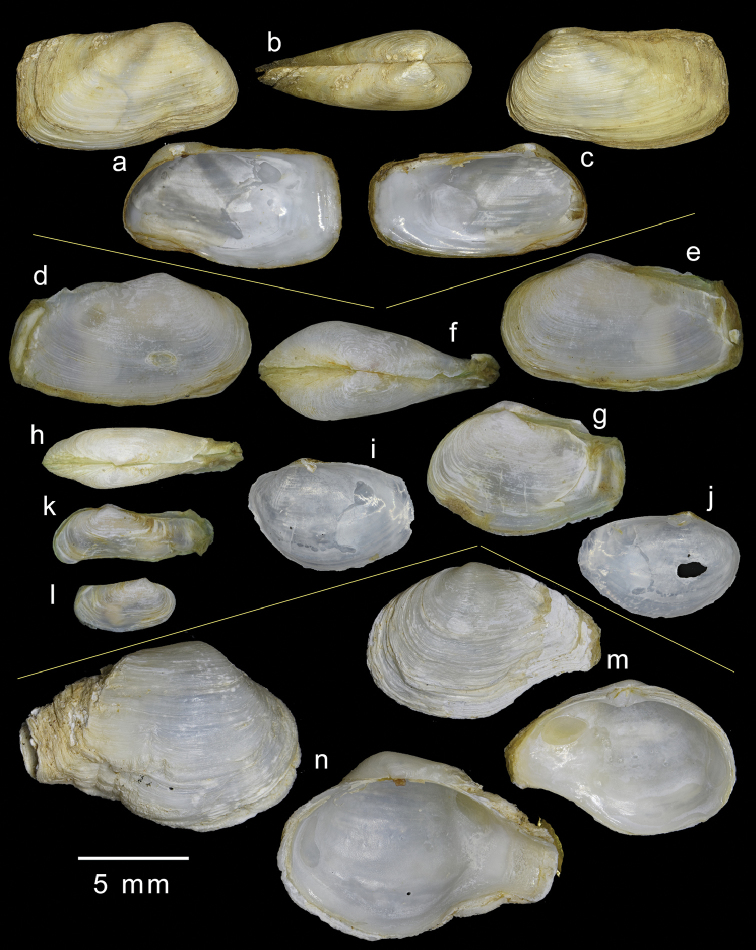
Comparison of shells of three species of epibyssate nestling species of *Sphenia*. **a–c***Spheniabinghami*, British Isle. **d–j***Spheniaperversa*, Kungkraben Bay, Thailand **m–n***Spheniaruepellii*, Yemen, Arabian Sea.

Of available myid genera, *Sphenia* is the most similar, and indeed EA Smith (1893) placed his Indian species *sowerbyi* in that genus. However, from comparisons within the species assigned to *Sphenia*, there is evidence that two groups are present; those species that are epibyssate (Fig. 4) and nestle among hard substrates (*S.binghami*, *S.perversa*, and *S.rueppelli*), and those that are endobyssate in soft substrates (brackish water taxa).

Given that we have both morphological and molecular distinctions between *Sphenia* sensu stricto and the endobyssate taxon, we describe the latter as a new genus, *Indosphenia*.

### Myoidea Lamarck, 1809

#### Myidae Lamarck, 1809

##### Indosphenia

Taxon classificationAnimaliaMyidaMyidae

Oliver, Hallan & Jayachandran gen. n.

http://zoobank.org/16D87E93-91A4-41E6-97E9-91903543A6DE

###### Type species.

(here designated) *Indospheniakayalum* sp. n.

###### Nominal species included.

*Spheniasowerbyi* EA Smith, 1893; *Corbulaalcocki* Preston, 1907; *Corbulagracilis* Preston, 1907; *Cuspidariaannandalei* Preston, 1915; *Cuspidariacochinensis* Preston, 1916.

By inference from descriptions *Corbulaabbreviata* Preston, 1907; *Corbulacalcaria* Preston, 1907; *Corbulapfefferi* Preston, 1907; *Corbulachilkaensis* Preston, 1911.

###### Description.

Slightly inequivalve, left valve smaller than right valve, almost equilateral to posteriorly extended, rather inflated. Outline subovate, anterior end broadly rounded; posterior narrowed, sub-rostrate. Sculpture of commarginal lines and very thin, weak lamellae; rostrum with a defined keel, at least in early growth stages. Pallial sinus very shallow, adductor muscle scars subequal, posterior scar subcircular, anterior scar elongate. Right valve with sub-umbonal, depressed resilifer accommodating chondrophore from left valve. Anteriorly, small projecting pseudo-tooth appears as extension of anterior margin (Fig. 3d). Left valve with projecting laminar chondrophore (Fig. 3d) plus shallow triangular depression anteriorly. Ligament attachment in narrow deep groove and extending over anterior part of chondrophore; ligament separated from posterior flange by weak ridge. Chondrophore extending posteriorly as narrow flange with median flexure in some individuals, its posterior end rounded and its outer face slightly sinuous in juveniles; in adults, posterior flange projects beyond ligamental portion and is rounded.

In larger specimens, anterior tooth on right valve can be eroded and scarcely visible, chondrophore can project further and flange can be reduced. Mantle edge fused except for pedal gape and short paired fused siphons. Mantle patterned with darkly pigmented radiating blotches. Gills with both demibranchs. Labial palps small. Byssus of very fine threads, but not observed in all species.

###### Etymology.

*Indosphenia* - combining the taxon provenance (India) with the related genus *Sphenia*. Gender feminine.

###### Remarks.

The molecular and morphological data strongly suggest that *Indosphenia* is a member of the Myidae. Huber (2010) postulated that all *Corbula* species described from Port Canning by Preston (1907) should be considered as a single species in the genus *Potamocorbula*. This conclusion was apparently reached on the basis of Preston’s descriptions rather than examination of actual specimens. Given that Preston did not describe the hinge and seemingly confused the anterior and posterior ends, it is not surprising that their true relationship went unrecognised until now.

The structure of the hinge of *Indosphenia* is generally identical to that of *Sphenia* sensu stricto as represented by its type species *S.binghami*, and its Indian Ocean counterpart *S.perversa*. However, this structure is also very similar to that seen in the juveniles of both *Myaarenaria* and *Myatruncata*. Consequently, hinge structure would not appear to be a useful character at the generic level. Instead, it suggests that *Sphenia* represents a neotenous retention of the juvenile byssate characters of *Mya*.

Compared to the deep burrowing habits of *Mya*, *Sphenia* exhibits a byssate nestling habit, which is manifested in their differing characters; typical *Sphenia* (Fig. 4) is irregular, heteromyarian and strongly inequilateral with the anterior end reduced. The posterior end is subtruncate and the siphons are large with well-developed musculature (Fig. 11e) and a corresponding deep and broad pallial sinus inside the shell valves. The following species share the nestling habit with corresponding similarities in shell form and anatomy: *S.binghami*, (see Yonge 1951), *S.perversa* (see this paper), *S.antillensis* Dall & Simpson, 1901 (see Narchi and Domaneschi 1993), *S.fragilis* (H & A Adams, 1854), *S.hatcheri* Pilsbry, 1899 (see Pastorino and Bagur 2011), *S.coreanica* Habe 1951 (see Zhang et al. 2012) and *S.elongata* Zhang et al., 2012. A further four East Pacific species are described by Coan (1999) and these too would appear to be nestling taxa.

*Indosphenia* differs from *Sphenia* in being almost equilateral with a narrow, almost rostrate, posterior end and a very short pallial sinus. This reflects the infaunal lifestyle of *I.kayalum*. Some specimens of *I.sowerbyi* have the posterior end encrusted with epifauna while the anterior end is relatively clean, suggesting that this species lives in sediment with the anterior end at, or close to, the sediment surface.

##### Indosphenia abbreviata

Taxon classificationAnimaliaMyidaMyidae

(Preston, 1907)

Corbula abbreviata Preston, 1907: 215, fig. 1; Ramakrishna and Dey 2010: 269.
Corbula alcocki Preston, 1907: 215, fig. 2.
Corbula calcarea Preston, 1907: 216, fig. 3; Ramakrishna and Dey 2010, 269.
Corbula gracilis Preston, 1907: 216, fig. 4; Ramakrishna and Dey 2010: 270.
Corbula pfefferi Preston, 1907: 216, fig. 5.
Potamocorbula abbreviata (Preston): Huber 2010: 771; Subba Rao 2017: 431; MolluscaBase 2018b.

###### Type material examined.

*Corbulaalcocki*, Syntype, 1 shell, Port Canning, purchased from Preston, HMUK1909.8.18.33.

*Corbulagracilis*, Syntype, 1 shell, Port Canning, purchased from Preston, HMUK1909.8.18.32.

The type material of *C.abbreviata*, *C.calcarea*, and *C.pfefferi* was not available for study.

###### Other material examined.

*Sphenia* sp. 20 shells and single valves, “Port Canning” [can be interpreted as the Matla River at Port Canning], SE of Kolkata, 22°18.7'N, 88°40.6'E, ex Godwin-Austin Coll, NHMUK 20170342.

*Sphenia* sp. 5 shells and 1 single valve, Pt. Canning, Matla River, ex Blandford Coll, HMUK 20170343.

*Corbulaintumescens* manuscript name of Stoliczka. 4 shells. Canning, ex. Dr F. Day, HMUK91.9.19.20-3.

*Corbulatumescens* manuscript name of Day 4 shells. Port Canning, NHMUK 88.12.4.703-6.

###### Type localities.

All species described by Preston (1907) are localised as “Port Canning, Lower Bengal; in brackish pools”. Port Canning lies on the Matla River approximately 80 km upstream from the mouth of the estuary. Both the manuscript names of Stoliczka have the same locality of “Port Canning”.

###### Description.

**Figure 5.**
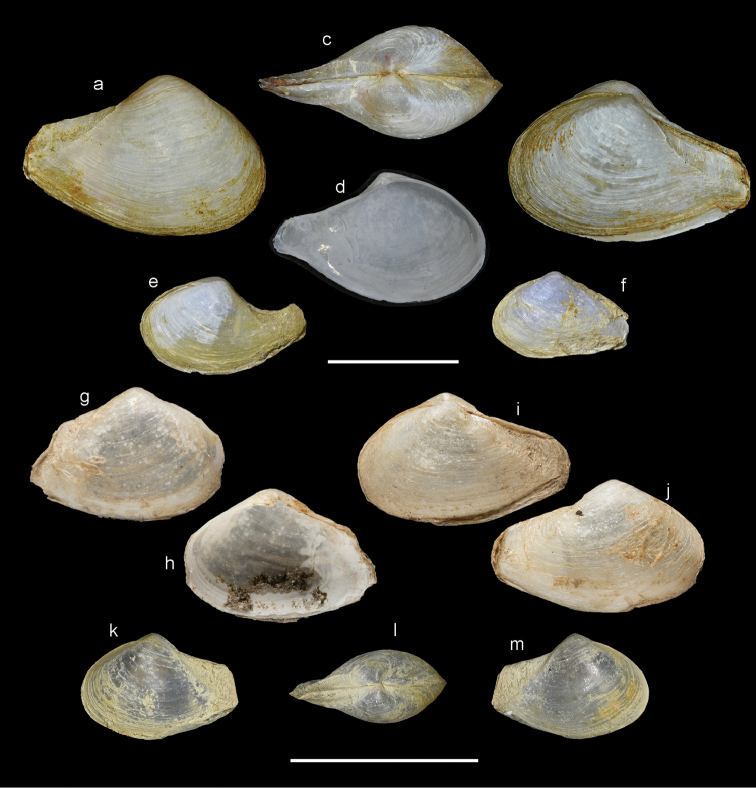
Shell variation in *Indospheniaabbreviata*. **a–d** typical form from Port Canning, NHMUK 20170342 **e, f** distorted varieties from Port Canning, NHMUK 20170342 **g, h** syntype of *Corbulaalcocki* Port Canning, NHMUK 1909.8.18.33 **i, j** syntype of *Corbulagracilis* , Port Canning NHMUK 1909.8.18.32 **k–m** syntype of *Cuspidariaannandalei* NMW1955.158.18922. Scale bar: 5 mm.

Shell (Fig. 5a–d) small, reaching 10 mm in length, thin, fragile, inequivalve - left valve slightly smaller; slightly inequilateral, beaks slightly to the front of centre (PL/AL = 1.3); moderately inflated (L/T = 2.1). Beaks prosogyrate, directed anteriorly. Outline subovate-sub-rostrate, L/H = 1.6, anterior end broadly rounded, ventral margin curved, posterior end much narrower than anterior end, sub-rostrate. Posterior area demarcated by sharp carina running from beak to ventral margin of rostrate posterior end. Postero-dorsal margin concave. Posterior end rounded, but often eroded and obscured by periostracum extending beyond shell. Sculpture of weak commarginal lines and raised threads forming weak projections on posterior carina.

Prodissoconch (Fig. 9b) consisting of small P1 (65 µm) and much larger P2 (182 µm); P1 with punctate micro sculpture; P2 with commarginal ridges crossed by sparse radial threads. Hinge myid, with chondrophore in left valve. Chondrophore (Fig. 7a) with proportionately narrow ligament insertion plate and long posterior flange; ridge between ligament area and flange weak. Right valve with very weak pseudo cardinal tooth (Fig. 7g) commonly rising dorsally to obscure beak.

###### Intraspecific variation.

**Figure 6.**
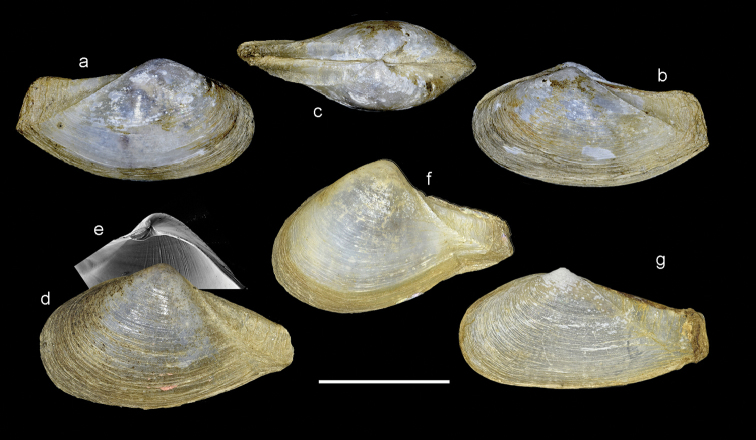
Variations of *Indospheniaabbreviata* all from Port Canning. **a, b** narrow form , NHMUK 20170343 **d–f***tumescence* form NHMUK 88.12.4.703 **e** Scanning electron micrograph of interior of **d** showing radial ridges **g***intumescence* form NHMUK 91.9.19.20. Scale bar: 5 mm.

**Figure 7.**
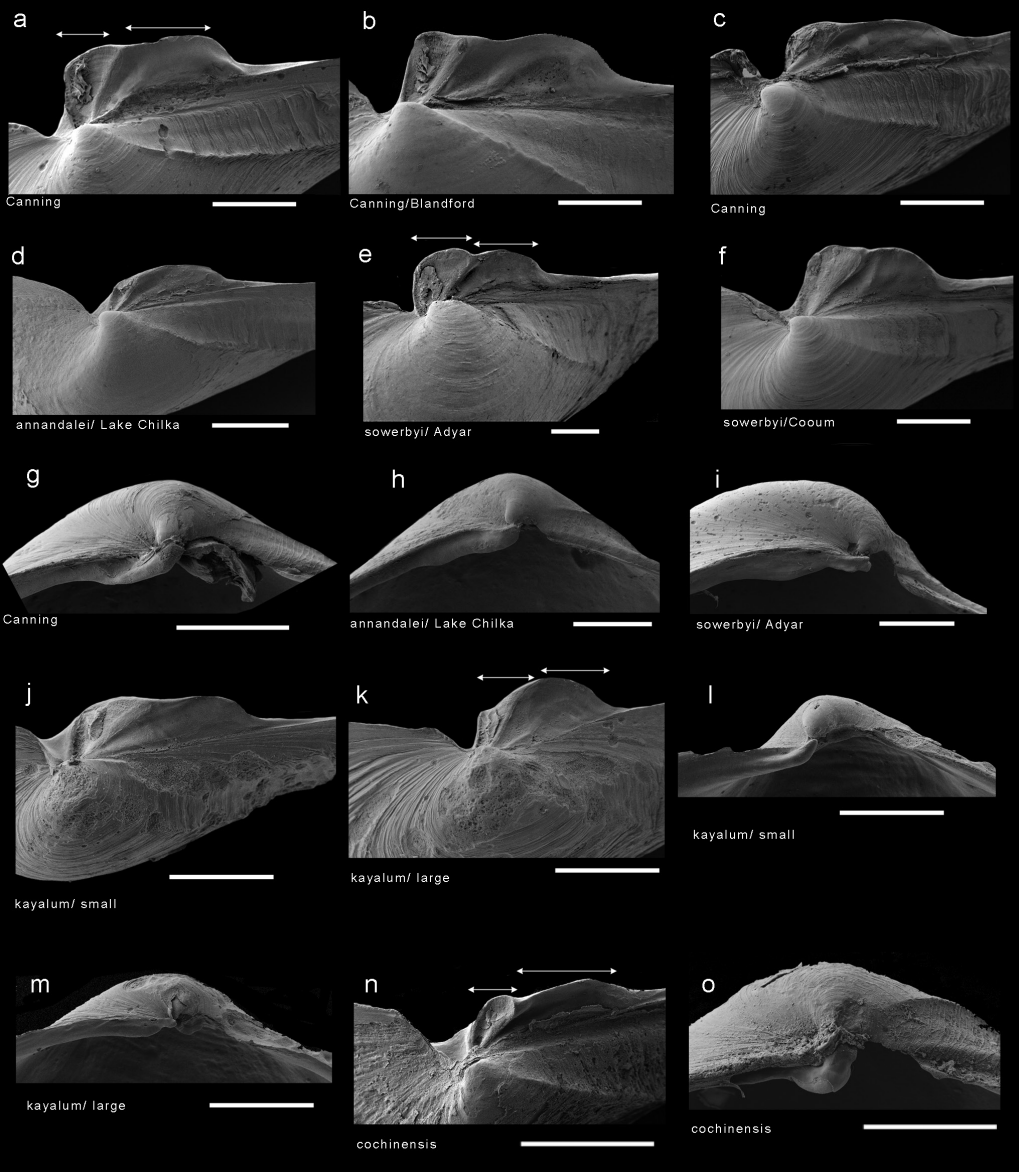
Scanning electron micrographs of the hinges of *Indosphenia* species. **a** chondrophore of *I.abbreviata* typical form from Port Canning **b** as **a** but from narrow form **c** as **a** but from distorted form **d** chondrophore of syntype of *Cuspidariaannandalei***e** chondrophore of *I.sowerbyi* from Adyar **f** as *e* but from Cooum **g** right valve pseudo tooth in *I.abbreviata***h** right valve pseudotooth in syntype of *Cuspidariaannandalei***i** right valve pseudotooth in *I.sowerbyi***j–k** chondrophores in small and large specimens of *I.kayalum***l–m** right valve pseudo tooth small and large specimens of *I.kayalum***n–o** chondrophore and right valve pseudo tooth of *I.cochinensis*. Scale bar: 5 mm.

Some shells in the unidentified samples from Port Canning are distorted with the rostrate posterior end upturned (Fig. 5e), while others are truncate posteriorly (Fig. 5f). While the overall outline is different, the hinge remains identical in structure (Fig. 7c). The syntype of *Corbulaalcocki* (Fig. 5g, h) represents the posteriorly truncated form, whereas the syntype of *C.gracilis* (Fig. 5i, j) is closer to the typical shape. The samples labelled as *Spheniatumescens* and the sample collected by Blandford, contain larger shells (up to 12 mm in length) (Fig. 6f). Of these, many are more elongate and narrower (Fig. 6a–d, g) and with a larger length to height ratio of 1.9, and where the posterior dorsal margin is less concave. Of the species described by Preston (1907), that of *calcarea* may be representative, but it is less than half the size of the shells described here. In one shell, from the *tumescens* sample, radial striations are present (Fig. 6e), most visible on the interior (Preston’s *pfefferi* form). The prodissoconch (Fig. 9a) is identical to that of the typical form in having a radial sculpture.

###### Remarks.

The shells from Port Canning are highly variable, with many showing distortions of some degree, although the overall form is broadly rounded with a deep anterior end and a sub-rostrate posterior end. Some samples contain a higher proportion of shells that are less deep and more elongate, and these have the manuscript names of *tumescens* Stolizcka and *intumescens* Day.

While the extremes of this form look very different, there are intergrades, and in all of these the prodissoconch has the same pattern of radial striations.

While Port Canning is given as the locality, the precise habitat is not provided for these samples, although one does mention the Matla River. Preston (1907) stated that his species came from brackish pools, so it may be that this species inhabits a range of habitats within the Matla River estuary. Port Canning is some 80 km from the open sea of the Bay of Bengal and is undoubtedly subject to much variation in salinity, certainly between isolated pools and the main river channel. Without supporting molecular data, we regard these forms to be part of a single species. The earliest available name for this taxon is *abbreviata* Preston, 1907, which is based on individuals that in the context of the current sample set may not be typical, in having the posterior end shortened. However, we consider that Huber (2010) was the first reviser of this group of species and we therefore retain the earliest name and the one given priority by him. This is also the name adopted by Subba Rao (2017).

##### Indosphenia abbreviata chilkaensis

Taxon classificationAnimaliaMyidaMyidae

(Preston, 1911)

Corbula chilkaensis Preston, 1911: 39, fig. 2; Subba Rao 2017: 432.
Cuspidaria annandalei Preston, 1915: 308, figs 23, 23a; Ramakrishna and Dey 2010: 303–4; MolluscaBase 2018d.

###### Type material examined.

*Corbulachilkaensis*. Type material was not available for study.

*Cuspidariaannandalei*. Syntypes, 3 shells, Lake Chilka [this can be interpreted as 19°42.9'N, 85°18.6'E]. Ex Preston, NMW.1955.158.18922-18923.

###### Type localities.

*Corbulachilkaensis*. “Rambha, S. end of Lake Chilka” [this can be interpreted as off Rambha approximately 19°31.2'N, 85°6.3'E].

*Cuspidariaannandalei*. “Lake Chilka, 4–9 miles E. by S.1/2 S. of Patsahanipur, 4–5 ft” [this can be interpreted as off Patasanipur approximately 19°42.9'N, 85°18.6'E].

###### Remarks.

Examination of shells (Fig. 5k–m) of *Cuspidariaannandalei* shows these to be identical with small specimens of the typical Port Canning form, including the presence of radial striations on the prodissoconch. Lake Chilka, some 55 km in length, is situated in the northern part of the Bay of Bengal approximately 400 km south-west of Port Canning and is largely isolated from the Bay of Bengal by a long shore bar, only connected by a channel less than 2 km wide. For the moment, and based on significant morphological similarities, we regard all of the taxa from the northern part of the Bay of Bengal to be a single species, taking the name of *Indospheniaabbreviata* (Preston, 1907), with the Lake Chilka population herein deemed a subspecies. However, we note that future molecular study may suggest genetic divergence not readily apparent based on shell morphology.

##### Indosphenia sowerbyi

Taxon classificationAnimaliaMyidaMyidae

(EA Smith, 1893)

Sphenia sowerbyi EA Smith, 1893: 280, pl. 15, fig. 8.

###### Type material examined.

Syntypes, 5 shells, Ariancoupar near Pondicherry [this can be interpreted as the Chunnambar River mouth, Ariyankuppam, Pudicherry, 11°52.8'N, 79°48.5'E]. NHMUK 1893.3.16.6-10.

###### Other material examined.

4 shells, NMW 1955.158 15753 and 50+ shells, NHMUK 1953.1.7, all from Adyar, Madras [this can be interpreted as the Adyar River Mouth, Adyar, S of Chennai], 13°0.8'N, 80°15.4'E, all Winckworth Collection, March 1931 to June 1936.

50+ shells, Cooum River, Madras [this can be interpreted as the Cooum River mouth, Chennai, 13°4.3'N, 80°16.1'E]. Winckworth Collection, September 1931.

###### Type locality.

Ariancoupan, Pondicherry [this can be interpreted as the Chunnambar River Mouth, Ariyankuppam, Pudicherry, 11°52.8'N, 79°48.5'E].

###### Description.

**Figure 8.**
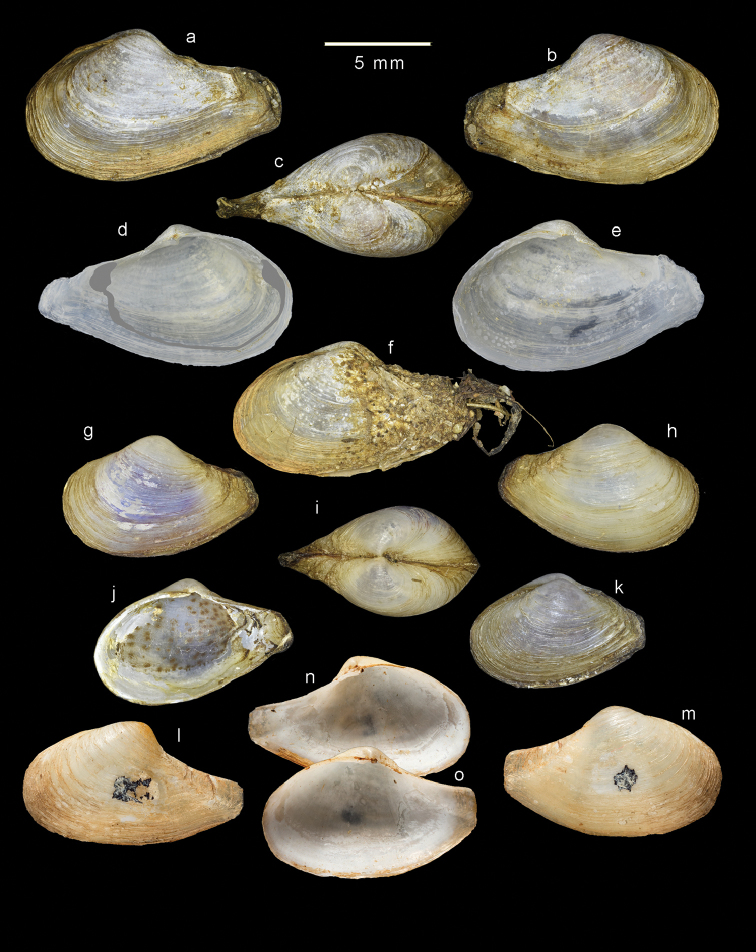
Variations of *Indospheniasowerbyi*. **a–c** typical form from Adyar river **d–e** internal views of cleaned valves from Adyar river, pallial line false colour added **d**. **f** specimen with epifauna attached to posterior end, from Adyar river **g–h** specimen from Cooum river **j** internal showing blotched pattern on dry mantle tissue **k** posterior sculptured variety from Cooum river **l–n** Syntype of *Spheniasowerbyi* from Ariancoupan, Pudicherry NHMUK 1893.3.16.6-7. Scale bar: 5 mm.

**Figure 9.**
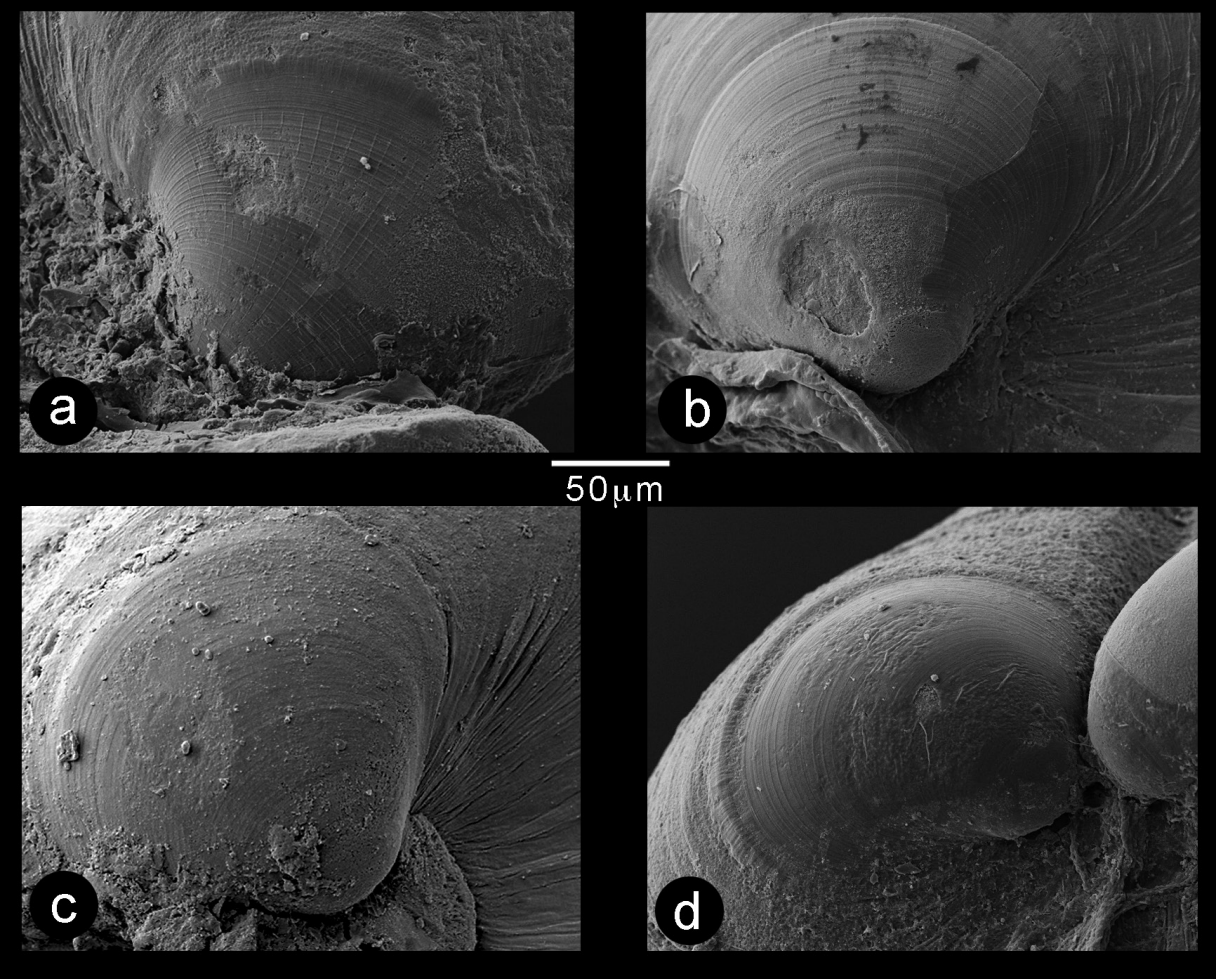
Scanning electron micrographs of the prodissoconchs of *Indosphenia* species. **a***I.abbreviata* elongate form **b***I.abbreviata* typical form **c***I.sowerbyi***d***I.kayalum*.

The sample from Adyar most closely matches the form of the syntypes (Fig. 8l–o) from Ariyankuppam, and is described here as typical. Shell (Fig. 8a–e) to over 12 mm in length, inequivalve, left valve slightly smaller; slightly inequilateral, beaks slightly to the posterior (PL/AL = 0.83–0.97); moderately inflated (L/T = 1.9– 2.2). Beaks prosogyrate, directed anteriorly. Outline subovate-sub-rostrate, anterior end broadly rounded, ventral margin curved, L/H = 1.6–1.8; posterior end much narrower than anterior end, sub-rostrate, demarcated initially by sharp carina running from beak to ventral margin of posterior end, but this carina rapidly becoming obsolete; posterior dorsal margin concave; posterior end rounded, often eroded and obscured by periostracum extending beyond shell. Sculpture of weak commarginal lines and dense, thin raised lamellae - these more prominent on angle of rostrum. Exterior of shell with persistent, thin, straw-coloured periostracum. Prodissoconch (Fig. 9c) small P1 (75 µm) much larger P2 (189 µm); P1 with punctate micro sculpture, P2 with commarginal ridges. Hinge myid, with chondrophore in left valve. Chondrophore (Fig. 7e) with proportionately wide ligament insertion plate and short posterior flange with rounded margin; ridge between ligament area and flange prominent. Right valve with very weak pseudo cardinal tooth (Fig. 7i) commonly rising dorsally to obscure beak. Muscle scars poorly defined. Anterior adductor muscle scar elongate and placed medially on anterior face. Posterior adductor muscle scar subcircular, placed close to dorsal margin. Pallial sinus a shallow depression, more sinusoidal than U-shaped (Fig. 8d).

###### Intraspecific variation.

Comparisons of shells from the Adyar and Cooum rivers show some significant difference in shape, despite the rivers being only 6 km apart. The shells from the Cooum River (Fig. 8g–i, k) are shorter posteriorly, thus the beaks are closer to the posterior end, and the height and tumidity are proportionately greater. The shells from the Cooum River are more robust and about 20% are tinged with pale purple (Fig. 8g). Although the tissues are dry, the blotched pattern (Fig. 8j) on the mantle has been preserved and it matches that present in both *I.abbreviata* and *I.kayalum*.

###### Remarks. *Indospheniasowerbyi*

has a thicker shell than either *I.abbreviata* or *I.kayalum* and possesses a much more prominent sculpture. The beaks lie closer to the posterior end, which is the opposite of both *I.abbreviata* and *I.kayalum*. Furthermore, the posterior carina is not developed, whereas in *I.abbreviata* it is finely developed. Additionally, prodissoconch 2 shows no radial sculpture, as is present in *I.abbreviata*, making it more similar to *I.kayalum* in this character.

##### Indosphenia kayalum

Taxon classificationAnimaliaMyidaMyidae

Oliver, Hallan & Jayachandran, gen. n. et sp. n.

http://zoobank.org/1AA6C5B9-A1B4-4671-A138-84AE3A7A181B

###### Type material.

**Figure 10.**
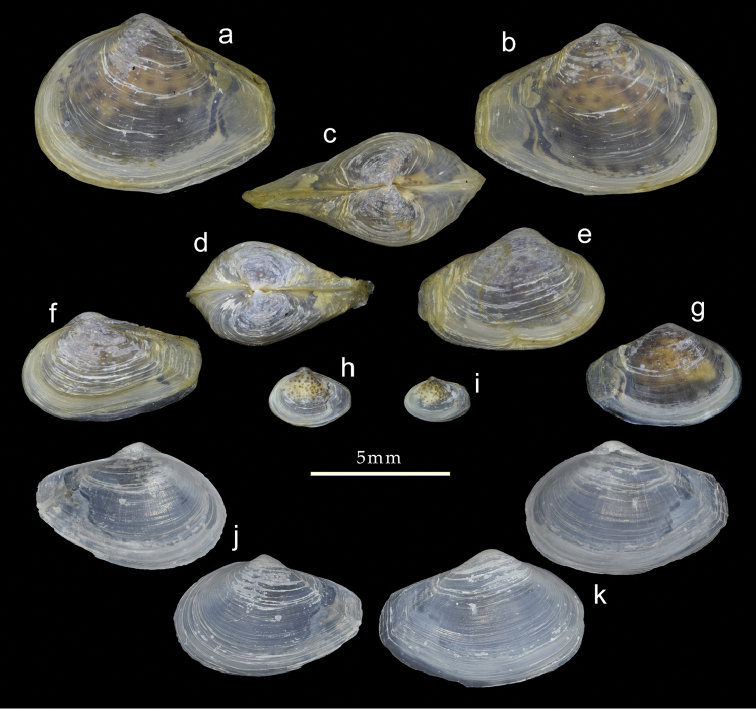
Specimens of *Indospheniakayalum*. **a–c** holotype **d–i** paratypes showing size series and variations **j, k** interior and exterior of cleaned shells.

**Holotype** (Fig. 10a–c), 1 specimen in 70% ethanol, Length = 7.6 mm. Anterior length = 3.4 mm, Height = 5.4 mm, Tumidity = 3.6 mm. Ezhupunna region of Cochin Backwater, Vembanad Lake 9°50'43.9"N, 76°17'17.2"E. Coll. Philomina Joseph, 3 March 2016. ZSI M-31827/8. **Paratypes** 20 specimens, as holotype, ZSI M-31828/8. Voucher specimens associated with GenBank sequences MH644188-644191, as holotype, ZSI M-31829/8 to M-31833/8

###### Type Locality.

Ezhupunna region of Cochin Backwater, Vembanad Lake 9°50'43.9"N, 76°17'17.2"E.

###### Description.

*Shell* (Fig. 10a–k) small, to 8.2 mm in length, thin, translucent, fragile, slightly inequivalve - left valve slightly smaller and overlapped by right valve, tumid across umbonal region (length/tumidity ratio = 2.26 but variable, some specimens more tumid, some much less, range from 1.8 to 2.6. Outline inequilateral, beaks slightly in front of midline with average ratio of posterior length/anterior length = 1.25, but variable due to degree of posterior extension, more equilateral in small shells; overall subovate, broadly rounded anteriorly, narrowed posteriorly, sub-rostrate. Poorly defined weak ridge demarcates narrow posterior dorsal area, best seen in juveniles. Sculpture of commarginal growth lines and weak (but raised) threads. Prodissoconch (Fig. 9d) small P1 (55 µm) much larger P2 (145 µm); P1 with punctate micro sculpture, P2 with commarginal ridges. Hinge myid with chondrophore in left valve. Chondrophore projecting, laminar (Figs 3d, 7j–k), with shallow triangular depression in front; ligament attachment in narrow, deep groove, extending over anterior part of chondrophore; ligament separated from posterior flange by weak ridge. Chondrophore extending posteriorly as narrow flange, with median flexure; posterior rounded and outer face slightly sinuous in juveniles (Fig, 7j), rounded in adults (Fig. 7k). Right valve (Fig. 3d, 7l–m) with resilifer, depressed subumbonally accommodating left valve chondrophore. Anteriorly small projecting pseudo- tooth appears as extension of anterior margin. Muscle scars poorly defined; anterior adductor scar elongate, placed medially on anterior face; posterior adductor scar subcircular, close to dorsal margin. Pallial sinus (Fig. 10j, k) shallow depression, more sinusoidal than U- shaped.

**Figure 11.**
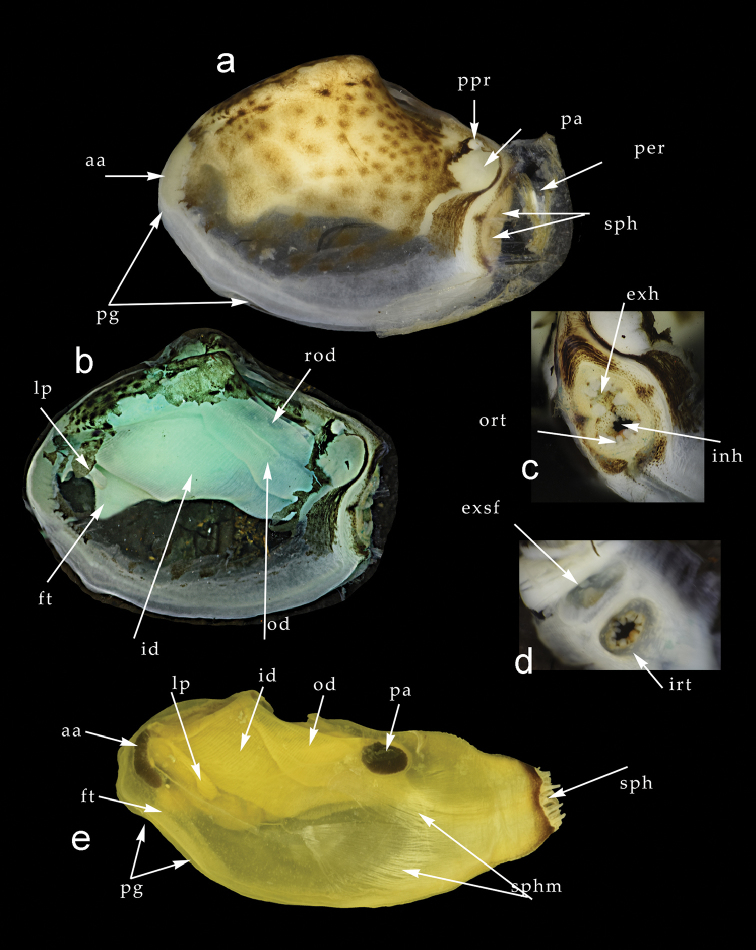
**a–d** Gross anatomy of *Indospheniakayalum***a** left side after removal of the shell **b** left side after removal of shell and mantle **c** exterior view of the siphonal apertures **d** interior view of the siphonal apertures **e** Gross anatomy of *Spheniabinghami* from the left side after removal of the shell and anterior mantle. Abbreviations: aa, anterior adductor muscle. exh, exhalant siphon. ft, foot. id, inner demibranch. inh, inhalant siphon. irt, inner ring of tentacles. lp, labial palps. od, outer demibranch. ort, outer ring of tentacles. pa, posterior adductor muscle. per, periostracum. pg, pedal gape. ppr, posterior pedal retractor muscle. rod, reflected portion of outer demibranch. sph, siphons. sphm, siphonal muscles.

*Anatomy* (Fig. 11a–d). Adductor muscles proportionately not large; posterior rounded in section, with posterior pedal retractor inserted immediately to its dorsal; anterior adductor muscle elongate, with anterior pedal retractor inserted at its dorsal edge. Mantle thin, distinctly patterned with brown radiating spots (Fig. 11a) visible through thin shell. Margins of mantle mostly fused, small anterior-ventral pedal gape present. Siphons (Fig. 11c, d) fused, rather short, with outer ring of 12–14 tentacles surrounding both inhalant and exhalant apertures; exhalent aperture with simple retractile tube, while inhalant aperture has inner ring of ten tentacles, the anterior-most larger and dull orange in colour. Foot small, distinct heel producing fine, multi-threaded byssus. Gills (Fig. 11b) large, inner and outer demibranchs present, fully reflexed; those of outer demibranch reflected dorsally. Labial palps very small with few sorting ridges. Only detail of alimentary canal readily observable is large style sac penetrating deep into posterior of foot.

###### Etymology.

The specific name *kayalum* is from “kayal” the Malayalam (South Indian language) name for the backwaters of Kerala state in which this species lives. The name is intended as a noun in apposition.

###### Habitat.

The specimens were found attached to the filamentous alga *Microspora* sp. that was growing on wooden poles in the shallow (1 m) channel in the backwater. The bottom substrate consisted of silty-sand and the measured salinity was 5‰ (oligohaline). Occurring with *I.kayalum* were the bivalves *Mytilopsissallei* and *Arcuatula* along with a large number of amphipods and polychaetes.

###### Remarks.

In overall shell shape, *Indospheniakayalum* is most similar to the typical form of *I.abbreviata*, but it differs in being less tumid and with a less well-defined rostrate posterior end on which the carina is weak to obsolete. Prodissococh 2 in *I.abbreviata* has radial lines, whereas in *I.kayalum* P2 has only commarginal lines. In size, the prodissoconchs are similar, as is also the punctate sculpture of P1. *Indospheniasowerbyi* is much larger, and more robust, such that the sculpture is of commarginal raised ridges, especially over the posterior area. In outline, *I.sowerbyi* is more tumid, less deep and the beaks are behind the midline. The sculpture of the larval shells is the same. *Indospheniacochinensis* has a heavier shell that is rather narrow, it has very prominent ridges, and is distinctly rostrate posteriorly.

##### Indosphenia cochinensis

Taxon classificationAnimaliaMyidaMyidae

(Preston, 1916)

Cuspidaria cochinensis Preston, 1916: 39, figs 17, 17a.
Cuneocorbula cochinensis (Preston, 1916): Oliver et al. 2017: 1224–5, fig. 2.

###### N.B.

**Figure 12.**
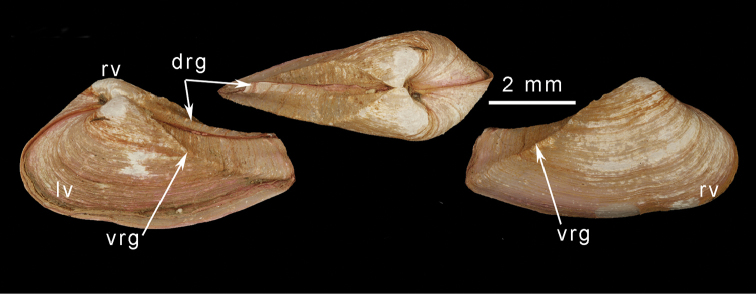
**a–c** Shell of *Indospheniacochinensis* from Kodungallur- Azhikode estuary. NMW.Z.2015.020.1.

*Cuspidariacochinensis* (Fig. 12a–c) was transferred from the Cuspidariidae into the corbulid genus *Cuneocorbula* by Oliver et al. (2016). Besides its extremely corbulid-like shell, the hinge is reminiscent of that of corbulids in which there is a relatively prominent tooth in the right valve. However, following this review, this tooth is no longer regarded as equivalent to the large anterior tooth possessed by *Potamocorbula* and *Lentidium*. The tooth in *C.cochinensis* (Fig. 7o) is a thickening of the hinge margin and thus structurally identical to the pseudo tooth seen in *Mya* and *Sphenia*. The chondrophore of *C.cochinensis* (Fig. 7n) has a posterior flange and this too is identical to the form seen in *Sphenia*. Consequently, we transfer *Cuneocorbulacochinensis* to *Indosphenia* in the Myidae.

###### Discussion.

The form of the hinge in *Indosphenia* is more similar to that of juvenile *Mya* and *Sphenia* supporting the placement of *Indosphenia* in the Myidae rather than the Corbulidae. The molecular phylogenetic analysis conducted herein, using COI places *Indospheniakayalum* in the Myidae, although with no significant bootstrap support. However, in combination with the morphological data, we consider this molecular result as adding further confidence to this family placement.

**Figure 13.**
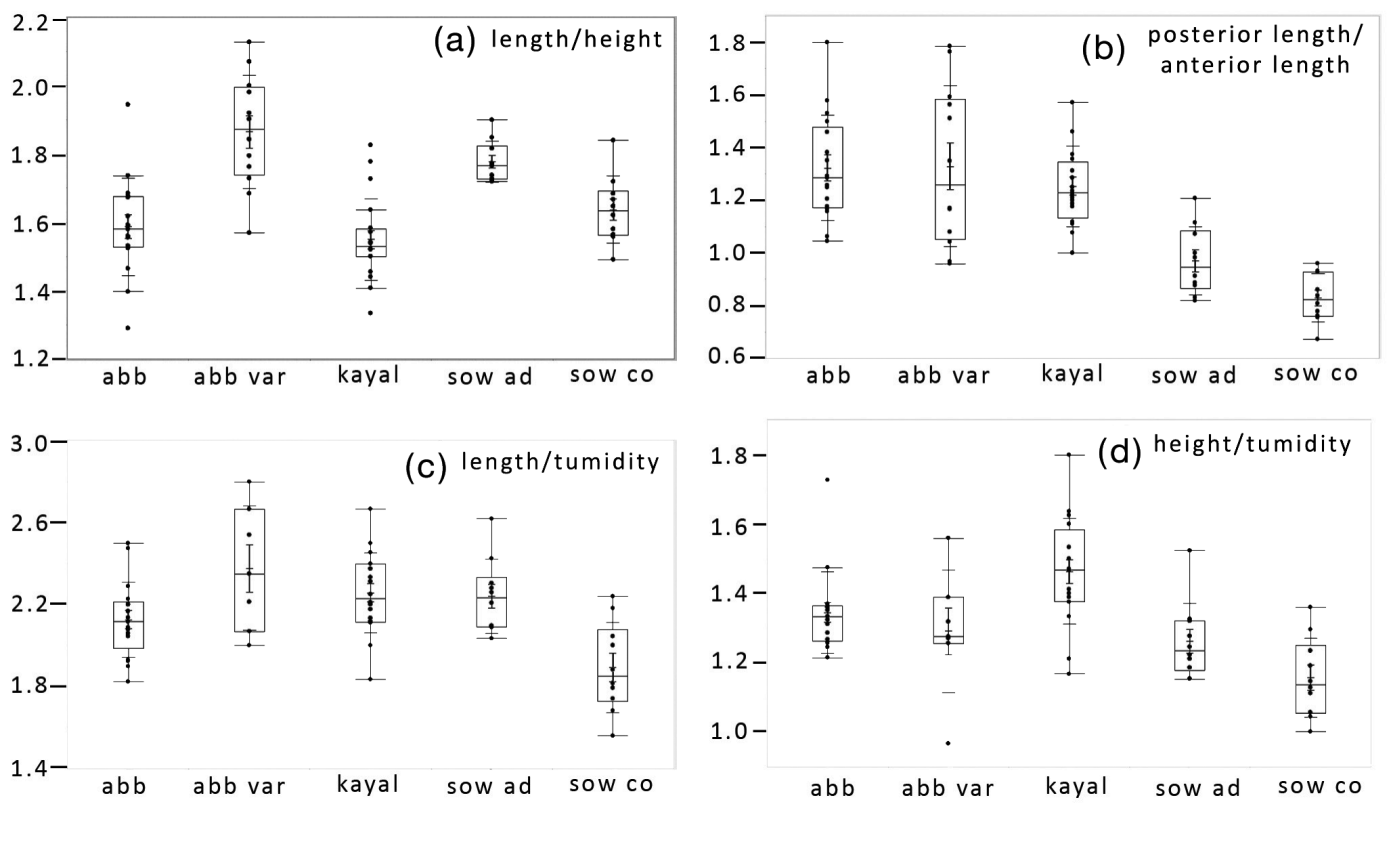
Box plots of the ratios of four parameters for shell shape in five populations of *Indosphenia*. **a** length to height **b** posterior length to anterior length **c** length to tumidity **d** height to tumidity. Abbreviations: abb, typical *I.abbreviata* in lot NHMUK 20170342. abb var, *I.abbreviata* variations in lots NHMUK20170343, NHMUK 91.9.19.20.& NHMUK 88.12.4.703. kayal, *I.kayalum*. sow ad, *I.sowerbyi* from Adyar. sow co, *I.sowerbyi* from Cooum.

The variability in shell form across the species herein assigned to *Indosphenia* can be seen both visually in the shells figured and in the graphical display of morphometric parameters (Fig. 13). The morphometric parameters show considerable variation within, and overlap between, most populations. Of the samples measured, that of *I.sowerbyi* from the Cooum River appears to be the most distinctive, even from the Adyar population of the same species only six kilometres distant. Using only dried shells from historical collections we cannot employ molecular techniques to distinguish any effects of geographical distance from habitat. In the Cochin Backwater, there are two quite distinct ecological forms that are separated ecologically. *Indospheniacochinensis* is infaunal, living in sand and found close to the entrance of the Kodungallur-Azhikode Estuary, where average salinity is 15.6 ‰. This contrasts with *I.kayalum* that lives byssally attached among algae and below in muddy sediment in an isolated embayment of Lake Vembanad, with a very low salinity of 5 ‰. These locations are approximately 40 km apart, but they are poorly connected, having separate channels into the Indian Ocean.

The environment may be more important than geographical separation in directing the form of the shell in *Indosphenia*, so although we have aggregated all of the Port Canning forms under *I.abbreviata*, it is possible that the two primary forms represent different species. The length/height and length/tumidity ratios (Fig. 13a, c) are significantly different but we have very poor locality and habitat details, only the general location of Port Canning, to make any inferences. Within the estuary of the Matla River there is likely to be a gradient of salinity and a variety of habitats. Taxonomic problems with brackish water faunas were raised by Muus (1967) and genetic divergence was reviewed by Cognetti and Maltagliati (2000) but studies do not reveal consistent results. The European brackish water cockle *Cerastodermaglaucum* (Bruguière, 1798) is morphologically variable, having received 44 species, subspecies, or varietal names (MolluscaBase 2018a). The variation was considered to be ecophenotypic, but a recent molecular investigation has shown that many populations are sufficiently genetically distinct to warrant some nomenclatural recognition (Tarnowska et al. 2010). A similar result was found for the bivalve *Mytilasterminimus* (Poli, 1795) (Camilli et al. 2001).

González-Wangüemert and Vergara-Chen (2014) studying *Pomatoschistusmarmoratus* (Risso, 1810) found significant diversity within Mar Menor lagoon, but Cavraro et al. (2017) did not find genetic diversity within the Venice lagoon for *Aphaniusfasciatus* (Valenciennes, 1821). Both studies found significant diversity between lagoon and marine populations of the same species. We cannot be certain if there is genetic diversity within the Port Canning samples, but it cannot be excluded.

The morphological complexity of *Indosphenia* suggests that the brackish waters of India may well represent fragmented habitats and as such would make excellent sites for the study of genetic isolation and divergence.

## Supplementary Material

XML Treatment for Indosphenia

XML Treatment for Indosphenia abbreviata

XML Treatment for Indosphenia abbreviata chilkaensis

XML Treatment for Indosphenia sowerbyi

XML Treatment for Indosphenia kayalum

XML Treatment for Indosphenia cochinensis
